# Valorization of *Arbutus unedo* L. Pomace: Exploring the Recovery of Bioactive Phenolic Compounds from Distillation By-Products

**DOI:** 10.3390/antiox14030278

**Published:** 2025-02-27

**Authors:** Ritamaria Di Lorenzo, Maria Grazia Ferraro, Ceferino Carrera, Federica Iazzetti, Nuria Chinchilla, Maria Maisto, María José Aliaño-González, Vincenzo Piccolo, Anabela Romano, Lucia Ricci, Bruno Medronho, Adua Marzocchi, Marialuisa Piccolo, Gian Carlo Tenore, Carlo Irace, Sonia Laneri

**Affiliations:** 1 Department of Pharmacy, University of Naples Federico II, Via D. Montesano 49, 80131 Naples, Italy; ritamaria.dilorenzo@unina.it (R.D.L.); federica.iazzetti@unina.it (F.I.); vincenzo.piccolo3@unina.it (V.P.); lucia.ricci@unina.it (L.R.); adua.marzocchi@unina.it (A.M.); marialuisa.piccolo@unina.it (M.P.); giancarlo.tenore@unina.it (G.C.T.); carlo.irace@unina.it (C.I.); slaneri@unina.it (S.L.); 2 Department of Molecular Medicine and Medical Biotechnology, University of Naples Federico II, 80131 Naples, Italy; mariagrazia.ferraro@unina.it; 3 Department of Analytical Chemistry, Faculty of Sciences, University of Cadiz, Agrifood Campus of International Excellence (ceiA3), Wine and Agrifood Research Institute (IVAGRO), 11510 Puerto Real, Spain; ceferino.carrera@uca.es (C.C.); bfmedronho@ualg.pt (B.M.); 4 Department of Organic Chemistry, Faculty of Sciences, University of Cadiz, Institute of Biomolecules (INBIO), 11510 Puerto Real, Spain; nuria.chinchilla@uca.es; 5 MED—Mediterranean Institute for Agriculture, Environment and Development, CHANGE—Global Change and Sustainability Institute, Faculdade de Ciências e Tecnologia, Campus de Gambelas, Universidade do Algarve, Ed. 8, 8005-139 Faro, Portugal; aromano@ualg.pt; 6 FSCN—Fibre Science and Communication Network Research Center, Surface and Colloid Engineering Department, Mid Sweden University, SE-851 70 Sundsvall, Sweden

**Keywords:** *Arbutus unedo*, pomace, by-product, cosmetic, polyphenols, antioxidant, skin cells

## Abstract

This study explores the potential of *Arbutus unedo* L. pomace, a by-product of the food industry, as a natural ingredient for skincare applications. In Portugal, *A. unedo* L. fruits are traditionally used to produce “Aguardente de Medronho”, a spirit with a protected geographical indication. The distillation process generates pomace, comprising skins, pulp remnants, seeds, and residual alcohol rich in phenolic compounds, whose levels are significantly increased during distillation. In addition to their documented high antioxidant content, these residues also display notable antimicrobial properties. However, their potential benefits for skin health have not yet been explored. The methodology entailed the preparation of the pomace extract and a comprehensive analysis of its polyphenolic content and antioxidant capacity under laboratory conditions and in preclinical cellular models. The by-products demonstrated a high polyphenol content and potent antioxidant activity, comparable to vitamin C. Bioscreening on human skin models (i.e., dermal fibroblasts and keratinocytes) revealed their ability to reduce reactive oxygen species (ROS) formation under oxidative stress in skin cells, highlighting their potential to mitigate skin aging and damage caused by environmental pollutants. Moreover, bioscreens in vitro revealed a high safety profile, without any interference with cell viability at concentrations up to 100 µg/mL. These findings support the use of *A. unedo* L. pomace extract as a sustainable ingredient for the development of antioxidant-rich and eco-friendly cosmetic or dermatologic products.

## 1. Introduction

In recent years, public awareness of skincare and its health implications has grown significantly, with particular attention on the harmful effects of aging and environmental pollutants [1]. This heightened consciousness has driven both consumers and the cosmetics industry to prioritize natural ingredients that are sustainable, organic, safe, and effective. To meet these demands, current research focuses on developing and validating natural compounds, such as polyphenol-rich extracts, polysaccharides, and organic acids. These compounds offer diverse benefits, including anti-aging, moisturization, anti-hyperpigmentation, and skin conditioning [2,3,4,5]. Consequently, the cosmetics industry has increasingly embraced the use of botanical extracts in its products, driving the exploration of ingredients with significant potential [6]. This shift towards natural and sustainable solutions reflects the growing consumer preference for environmentally friendly and health-conscious products [7,8,9]. These efforts align with the principles of a circular and sustainable economy, where even by-products such as distillation residues and production waste are increasingly recognized as valuable materials for diverse applications, including cosmetics.

One promising candidate is *Arbutus unedo* L., a small tree or shrub from the *Ericaceae* family native to the Mediterranean region and prevalent in countries like Portugal and Spain [10]. The fruits of *A. unedo* L. are renowned for their sweet taste when fully ripe and are rich in sugars, fatty acids, vitamin C, fibers, and polyphenols [11,12]. Polyphenols have been linked to a wide range of biological and health-related properties, including antibacterial, antiparasitic, antifungal, antidiabetic, antihypertensive, antiaggregant, antitumoral, anti-inflammatory, and antioxidant effects [13,14,15,16]. Notably, they show biomedical potential in treating conditions such as kidney, gastrointestinal, dermatologic, urological, cardiovascular, and hypertensive diseases [14,17,18,19,20,21].

However, due to their high sugar content and delicate texture, these fruits are highly perishable, limiting their use in fresh form [22]. In Portugal, *A. unedo* L. fruits are traditionally used to produce a protected geographical indication distillate known as “Aguardente de Medronho”, a popular aromatic spirit with an alcohol content of up to 50%, characterized by fruity flavors with hints of caramel, hazelnuts, and rosemary.

The distillation process generates a significant residue called “pomace”, comprising skins, pulp remnants, seeds, and residual alcohol. Approximately 7 kg of medronho fruit are required to produce one liter of distillate. A study performed by Alexandre et al. [23] demonstrated that this pomace contains phenolic compounds such as pyrogallol, gallic acid, catechol, and protocatechuic acid. Intriguingly, research by Rodrigues et al. [24] revealed that fermentation and/or distillation significantly increases the total flavonoid content in pomace, doubling the amount found in the raw fruit extracts.

It is hypothesized that several biochemical mechanisms may contribute to the observed increase in flavonoid content in *A. unedo* pomace after fermentation and distillation. A key process is the enzymatic deglycosylation of flavonol glycosides, mainly derived from quercetin, by yeast-derived β-glucosidase activity during fermentation [25]. This enzymatic reaction hydrolyzes the glycosidic bonds, resulting in the release of flavonoid aglycones, which are more bioavailable and have a higher antioxidant potential. Furthermore, it has been demonstrated that fermentation-induced microbial metabolism can trigger structural modifications, including methylation, oxidation, and hydrolysis, resulting in the generation of novel flavonoid derivatives or the enhancement of the solubility of pre-existing ones [26]. The distillation process has been shown to enhance the concentration of these flavonoids by virtue of its capacity to remove volatile components, thereby enriching the pomace in polyphenolic compounds [27].

Additionally, Duarte et al. [28] described the antioxidant and antimicrobial properties associated with this distillate. Given their rich chemical profile, *A. unedo* L. distillate residues represent an attractive candidate for cosmetics applications, particularly in products with antioxidant or anti-aging properties.

Therefore, the present study aims to evaluate the presence of bioactive compounds, especially polyphenols in *A. unedo* L. distillate residues, and explore their potential application in cosmetics or dermatology, investigating their ability to protect epidermal keratinocytes and dermal fibroblasts by ROS, which are considered to be the one of major cause of aging [29,30,31].

By exploiting this underutilized resource, the current research supports sustainable innovation favoring the circular economy and mitigating environmental pollution, while highlighting its significant potential in the field of skincare.

## 2. Materials and Methods

### 2.1. Sample

The pomace of *A. unedo* L. was acquired from a small distillery located in Monchique, Faro, Portugal. Initially, the distillation commenced with the harvested fresh fruits being meticulously sorted, involving the removal of peduncles, leaves, and any underdeveloped fruits. Subsequently, the sorted fruits were placed in a plastic barrel, with a specific volume of water added (typically, up to 3 L/15 kg of fruits). The barrels used for fermentation were hermetically sealed to minimize undesired oxidation. Notably, the alcoholic fermentation of *A. unedo* L. fruit occurred spontaneously, facilitated by naturally present yeasts. Upon completion of fermentation (lasting up to 2 months), the fermented fruits and liquid were transferred to a traditional copper-based distillation apparatus (with a maximum capacity of 120 L) to commence the distillation process. Throughout, close attention was paid to controlling the alcohol content to attain the desired percentage. Following the completion of the distillation, the *A. unedo* L. pomace was discarded, and the copper-distillation equipment underwent thorough cleaning in preparation for subsequent runs. The gathered pomace was then subjected to freezing at −20 °C and subsequently underwent lyophilization to eliminate all residual water content. The freeze-drying procedure was carried out utilizing a VirTis Benchtop Pro Freeze Dryer (SP Industries, Inc., Warminster, PA, USA). Post-lyophilization, the samples underwent further grinding utilizing an electric MKM6003 coffee grinder (BSH Electrodomésticos España S.A., Zaragoza, Spain). The resultant powder was stored at −20 °C until required for subsequent applications.

### 2.2. Chemicals

Methanol (MeOH) and formic acid of analytical grade were acquired from Thermo Fisher Scientific (Waltham, MA, USA) and Merck (Darmstadt, Germany), respectively. Milli Q water was acquired from a Millipore water purification system (Bedford, MA, USA). To adjust the pH levels of the extraction solvent, 1 M HCl and 0.5 M NaOH solutions from Panreac (Barcelona, Spain) were employed. Salts, reagents, gallic acid (purity ≥ 98% HPLC), catechin (purity ≥ 98% HPLC), quercetin (purity ≥ 98% HPLC), quercetin 3-*O*-glucoside (purity ≥ 98% HPLC), *p*-coumaric acid (purity ≥ 98% HPLC), and chlorogenic acid (purity ≥ 95% HPLC) were purchased from Sigma-Aldrich Chemical Co. (St. Louis, MO, USA).

In order to evaluate the antioxidant activity, 2,2-diphenyl-1-picrylhydrazyl (DPPH) and 2,2′-azino-bis(3-ethylbenzothiazoline-6-sulfonic acid) (ABTS) from Sigma-Aldrich (St. Louis, USA), and 6-hydroxy-2,5,7,8-tetramethylchroman-2-carboxylic acid (Trolox) from Thermo Fisher Scientific (MA, USA), were employed.

### 2.3. Polyphenol Identification and Quantification

The extraction of polyphenols from the *A. unedo* L. pomace samples was conducted as reported by Piccolo et al. [32] with slight modifications. In this method, the sample (1 g) was mixed with 5 mL of 80% aqueous methanol with 1% formic acid, vortexed for 1 min, and sonicated (continuous operative mode, 150 W Power, 40 kHz Frequency; Branson Fisher Scientific 150E Sonic Dismembrator) for 10 min. The mixture was centrifugated at 9000 rpm for 10 min and the supernatant was moved into another plastic tube. The pellet was subjected to another extraction cycle with 5 mL of solvent as previously described. The supernatants obtained were mixed, filtered with a 0.22 µm nylon filter (CellTreat, Shirley, MA, USA), and stored at −20 °C until analysis.

#### 2.3.1. Qualitative Polyphenol Analysis by HPLC–DAD–HESI–MS/MS

The qualitative polyphenol analysis was performed using a Dionex UltiMate 3000 HPLC system (Thermo Fisher Scientific, San Jose, CA, USA), equipped with an autosampler, a binary solvent pump, a diode-array detector (DAD), and an LTQ XL mass spectrometer (Thermo Fisher Scientific, San Jose, CA, USA). The chromatographic procedure followed Piccolo et al. [32] with minor modifications. The separation parameters were a column temperature of 35 °C, an injection volume of 5 µL, and a flow rate of 0.35 mL/min. A Kinetex^®^ C18 column (75 mm × 2.1 mm, 2.6 µm; Phenomenex, Torrance, CA, USA) was used for separation. The mobile phase consisted of 0.1% formic acid in water (A) and 0.1% formic acid in acetonitrile (B). The elution profile was as follows: 5% (B) held for the first 1.5 min, increasing from 5% to 95% (B) over 20 min, then 95% (B) within 5 min, with a 5 min maintenance, followed by a 3 min re-equilibration to the initial conditions. For the mass spectrometry, a heated electrospray ionization (HESI) source was used in negative ion mode with full scanning (FS) and data-dependent acquisition (DDA). Phenolic acids, hydroxycinnamic acids, flavanols, and flavanones were detected at 280 nm, while flavonols were detected at 360 nm. Fragmentation of ions was induced using argon with a collision energy of 35.0 eV. The source conditions were set as follows: sheath gas flow rate of 30, auxiliary gas flow rate of 10, capillary temperature of 320 °C, source temperature of 150 °C, source voltage of 3.5 kV, source current of 100 µA, capillary voltage of 31 V, and tube lens voltage of 90 V. DDA parameters were set as follows: MS scan range of 100–2000 *m*/*z*, minimum signal threshold of 500 counts, minimum MS signal count of 5, default charge state of 1, repeat count of 1, repeat duration of 20 s, exclusion list size of 50, and exclusion duration of 15 s.

#### 2.3.2. Quantitative Polyphenol Analysis by HPLC–DAD–FLD

The quantitative polyphenol analysis was performed using a Jasco Extrema LC-4000 HPLC system (Jasco Inc., Easton, MD, USA), coupled with an autosampler, a binary solvent pump, a DAD, and a fluorometric detector (FLD) used for the analysis. The chromatographic analysis was performed according to Maisto et al. [33], with slight modifications. A Kinetex^®^ C18 column (250 mm × 4.6 mm, 5 μm; Phenomenex, Torrance, CA, USA) was used for the chromatographic elution. The mobile phases were water acidified with 2% formic acid (A) and acetonitrile:water 50:50, *v*/*v* acidified with 0.5% formic acid. The separation method was performed according to the following conditions: 0–2 min, isocratic on 10% phase B; 2–52 min, linear gradient from 10 to 55% B; 52–62 min, linear gradient from 55 to 95% B; 62–70 min, isocratic on 10% B for column recondition. The chromatographic parameters were as follows: a column temperature of 30 °C, an injection volume of 20 µL, and a flow rate of 1 mL/min. Phenolic acids and hydroxycinnamic acids were evaluated at 280 nm, while flavonols were evaluated at 360 nm. Flavanol monitoring was performed using a fluorescence detector, with an excitation wavelength of 272 nm and an emission wavelength of 312 nm. Peak compounds were identified using the approach of standard addition to the samples, and the quantification was performed using calibration curves (R^2^ ≥ 0.99) at six different concentration levels (concentration range: 0.5–0.0005 mg/mL) and triplicate injections at each point.

#### 2.3.3. Total Polyphenol Content (TPC)

The total phenolic content (TPC) was determined by the Folin–Ciocalteau method according to Piccolo et al. [32]. A volume of 125 µL of the sample was mixed with 0.125 mL of Folin–Ciocalteau reagent, 1.25 mL of an aqueous solution of Na_2_CO_3_ 7.50 (*w*/*v*), and 1.50 mL of water (final volume of 3 mL). After 10 s of mixing, the solutions were incubated in the dark for 90 min. Later, the absorbance was evaluated at 760 nm with a V-730 UV–visible/NIR spectrophotometer operated by Spectra Manager™ Suite (Jasco Inc., Easton, MD, USA). The calibration curve was prepared using six concentrations of gallic acid (R^2^ ≥ 0.99), with concentrations ranging from 7.5 to 250.0 ppm, with a dilution factor of 1:2 and three replicates for each concentration. The extracts were analyzed in triplicate and the total phenolic content was expressed as mg gallic acid equivalents (GAE)/g dry weight (DW).

### 2.4. Antioxidant Activity

#### 2.4.1. DPPH Assay

The DPPH (radical 2,2-diphenyl-1-picrylhydrazyl) assay was used to measure the sample’s antioxidant activity [34]. The standard protocol involved the addition of 200 µL of hydroalcoholic extract to 1000 µL of a 0.05 mM DPPH methanolic solution. After mixing, the solutions were kept in the dark for 10 min. Once the reaction time was complete, the decrease in absorbance was monitored at 517 nm with a V-730 UV–visible/NIR spectrophotometer controlled by Spectra Manager™ Suite (Jasco Inc., Easton, MD, USA). The absorbance of methanolic DPPH solution without the sample (the control) was used as the blank. The inhibition percentage was then determined using the following formula:

% inhibition = [(1 − A_f_)/A_c_] × 100

where A_f_ represents the absorbance after 10 min, and A_c_ is the absorbance of the control at time zero. 6-Hydroxy-2,5,7,8-tetramethylchroman-2-carboxylic acid (Trolox) was used as the standard antioxidant. A calibration curve was created using eight concentrations ranging from 5 to 250 µM, with dilution factors of 1:2 and 1:5 and three replicates for each concentration. The extract was analyzed in triplicate, and the results were reported as µmol Trolox equivalent (TE)/g DW. Additionally, the IC_50_ values were reported, which represent the sample concentration required to reduce the initial ABTS concentration by 50%. The calibration curve concentrations for IC_50_ determination were 60, 120, 310, 630, 1250, 1900, and 1550 mg/L.

#### 2.4.2. ABTS Assay

The ABTS (2,2′-azinobis(3-ethylbenzotiazoline-6-sulfonate)) assay was used to measure the sample’s antioxidant activity according to Ratnavathi’s method [35]. The standard protocol involved the preparation of ABTS working solution by mixing 2.5 mL of ABTS solution 7.0 mM with 0.044 mL of potassium persulfate solution 140 mM, and incubating it in the dark at 5 °C for 7 h. Ethanol was used to dilute the solution until an absorbance value of 0.70 ± 0.05 at 754 nm (Jasco Inc., Easton, MD, USA). Later, hydroalcoholic extract (0.10 mL) was mixed with ABTS ethanolic solution (1 mL). The samples were incubated in the dark for 2.5 min and the decreasing absorbance was monitored at 517 nm. The absorbance of the ABTS ethanolic control solution was used as the blank. The inhibition percentage was determined using the following formula:

% inhibition = [(1 − A_f_)/A_c_] × 100

where A_f_ represents the absorbance after 10 min, and A_c_ is the absorbance of the control at time zero. Trolox was utilized as the standard antioxidant. A calibration curve was created using eight concentrations ranging from 5 to 200 µM, with dilution factors of 1:2 and 1:5, and three replicates for each concentration. The extract was analyzed in triplicate, and the results were reported as µmol Trolox equivalent (TE)/g DW. Additionally, the IC_50_ values were reported, which represent the sample concentration required to reduce the initial ABTS concentration by 50%. The calibration curve concentrations for IC_50_ determination were 60, 120, 310, 630, 1250, 1900, and 1550 mg/L.

#### 2.4.3. FRAP Assay

The FRAP (ferric reducing antioxidant power) assay was used to measure the sample’s ferric reducing activity [36]. The standard protocol involved the preparation of a 10 mM TPTZ (2,4,6-tris(2-pyridyl)-s-triazine) working solution by dissolving 33.5 mg of the reagent in 10 mL of 40 mM HCl solution. The FRAP working solution was prepared by combining 1 mL of TPTZ solution with 1 mL of 20 mM ferric chloride solution and 10 mL of 0.3 M acetate buffer at pH 3.6. The solution was preheated to 37 °C. Later, hydroalcoholic extract (0.15 mL) was mixed with FRAP working solution (2.85 mL), and the samples were kept in the dark for 4 min at 37 °C. After this period, the increase in absorbance was monitored at 593 nm with a V-730 UV–visible/NIR spectrophotometer controlled by Spectra Manager™ Suite (Jasco Inc., Easton, MD, USA). The absorbance of blank FRAP solutions, which contained no sample, was measured and subtracted from those of the test samples. Trolox was used as the standard antioxidant, and the results were reported as µmol TE/g DW. A Trolox calibration curve was developed with eight concentrations ranging from 5 to 200 µM, using dilution factors of 1:2 and 1:5, with three replicates per concentration.

### 2.5. Preclinical Models of Human Skin

HDFa cells, human primary adult dermal fibroblasts, were obtained from the skin of a White male donor (PCS-201-012™) and purchased from ATCC (University Boulevard, Manassas, VA, USA). HDFa cells were cultured in fibroblast basal medium (ATCC) supplemented with fibroblast growth kit–low serum (ATCC) containing recombinant human fibroblast growth factor (rh FGF, 5 ng/mL), L-glutamine (7.5 mM), ascorbic acid (50 µg/mL), hydrocortisone hemisuccinate (1 µg/mL), rh insulin (5 µg/mL) and fetal bovine serum (FBS, 2%). Moreover, penicillin–streptomycin–amphotericin B solution (penicillin: 10 units/mL, streptomycin: 10 µg/mL, amphotericin B: 25 ng/mL) was added. HDFa cells were seeded at a density of 2.5–5 × 10^3^ cells/cm^2^ and were passed when approximately 80% to 100% confluence was reached and only if cells were actively proliferating. The cells were kept at 37 °C in a humidified atmosphere containing 5% CO_2_, as per the ATCC manufacturers [37].

HaCaT cells, human immortalized keratinocytes (kindly provided by Dr. Valeria Cicatiello at the Italian National Research Council (CNR), Institute of Genetics and Biophysics, Naples, Italy) were maintained in a humidified 5% CO_2_ atmosphere at 37 °C and grown in DMEM (Invitrogen, Waltham, MA, USA) supplemented with 10% fetal bovine serum (FBS, Cambrex), L-glutamine (2 mM), penicillin (100 units/mL, Sigma-Aldrich), and streptomycin (100 μg/mL). Cells were seeded at a density of 2–4 × 10^4^ cells/cm^2^ and cultured to about 80–90% confluence [38]. Both HDFa and HaCaT cells provide ideal in vitro skin models to study biocompatibility and toxicological cellular responses.

### 2.6. Cellular Responses to In Vitro Treatments

The biological effects and cytotoxicity of *A. unedo* L. pomace extracts were investigated through the estimation of a “cell survival index”, arising from the combination of cell viability evaluation and automatic cell count [39]. Cells were inoculated in 96-microwell culture plates at a density of 10^4^ cells/well and allowed to grow for 24 h. The medium was then replaced with fresh medium, and the cells were treated for 48 h or 72 h with a range of concentrations (5 → 100 μg/mL) of *A. unedo* L. pomace extracts. DMSO was used as a vehicle to solubilize the extract powder, and then the final solution was filtered. DMSO toxicity was evaluated in control cultures at a concentration ranging from 0.025 to 0.5% *v*/*v* (the same concentrations used in bioscreen experiments) to exclude interference with cell viability. Cell viability was evaluated by the 3-(4,5-dimethyl-2-thiazolyl)-2,5-diphenyl-2H-tetrazolium bromide (MTT) assay procedure, which is a colorimetric assay based on living mitochondria’s capacity to convert the yellow MTT solution into insoluble purple formazan, evaluating cellular mitochondrial dehydrogenase activity levels. The assay was performed according to the manufacturer’s instructions (475989 Sigma-Aldrich). The absorbance was measured at 550 nm by using a microplate reader (Thermo Fisher). Cell number was determined by a TC20 automated cell counter (Bio-Rad, Milan, Italy), providing an accurate and reproducible total count of cells and a live/dead ratio in one step by a specific dye (trypan blue) exclusion assay [40]. The assay was performed according to the manufacturer’s instructions (1450021 Bio-Rad, San Diego, CA, USA).

### 2.7. ROS Detection In Vitro

In order to evaluate the antioxidant effect in vitro of *A. unedo* L. pomace extracts, we performed a highly sensitive ROS detection with a specific assay kit (CA0093 Canvax, Valladolid, Spain). To this end, HDFa and HaCaT cells were plated on a black 96-microwell culture plate at a density of 10^4^ cells/well. Following 24 h of growth, the medium was replaced with fresh medium and 50 μM of dichlorodihydrofluorescein–diacetate (H_2_DCF–DA) was added to the cells. By passive diffusion into the cell, the lipophilic H_2_DCF–DA undergoes cleavage of acetyl groups by cytosolic esterases, resulting in the formation of a non-fluorescent compound that is rapidly oxidized by ROS into the fluorescent 2′,7′-dichlorodihydrofluorescein (DCFDA). Then, cells were washed and treated with 50 μM of H_2_O_2_ to induce oxidative stress (a), in the presence of different concentrations (25, 50 and 100 μg/mL) of *A. unedo* L. pomace extract (b), or with 50 μg/mL of vitamin C, herein used as the reference antioxidant (c). The fluorescence intensity was detected after 24 h and 48 h by using a fluorescence microplate reader (Promega GloMax, Promega Italia, Milan, Italy) at Ex/Em = 485/530 nm, in accordance with the supplier’s specifications. The intensity of the fluorescent signal was proportional to the cytosolic ROS levels. Basal ROS production in untreated control cells was assigned a value of 100%.

### 2.8. Statistical Analysis

Quantitative analyses were carried out using GraphPad Prism version 9.0 for Windows (GraphPad Software, San Diego, CA, USA) using one-way ANOVA followed by Tukey’s post hoc test. Significant differences were accepted at the 95% confidence level. All analyses were performed in triplicate. The concentration–response curve, by nonlinear regression, was obtained using a curve-fitting program and was expressed as mean ± SEM (*n* = 15) of three independent experiments.

## 3. Results and Discussion

### 3.1. Polyphenol Characterization of A. unedo L. Pomace

#### 3.1.1. Qualitative Polyphenol Analysis

**Figure 1:**
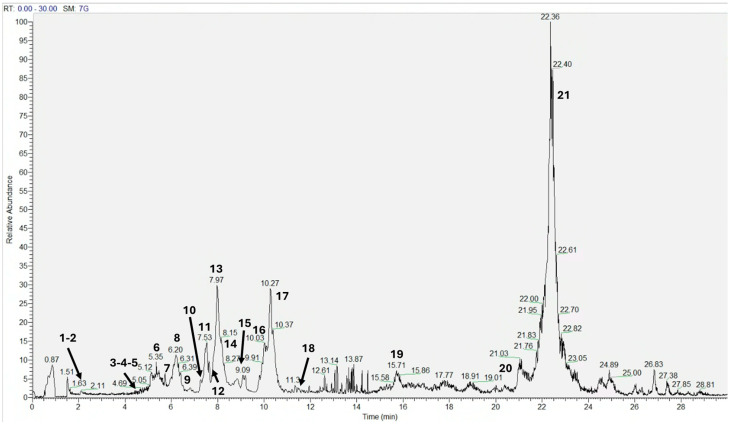
Chromatographic profile of *A. unedo* L. pomace extract in negative acquisition mode by HPLC–HESI–MS/MS analysis.

The sample of *A. unedo* L. pomace was extracted with a hydroalcoholic mixture for polyphenol analysis and the evaluation of antioxidant properties. Moreover, polyphenols determine antioxidant effects, which are beneficial for skin wellness and are suitable for the preparation of cosmetics and personal care products. The qualitative polyphenol analysis of *A. unedo* L. hydroalcoholic extract was performed by HPLC–DAD–HESI–MS/MS in negative acquisition mode (Figure 1). Based on a comparison with the literature data, fifteen compounds were putatively identified. The retention time, the quasi-molecular ion (*m*/*z*), and the fragment ions are listed in Table 1. Base peak ions are shown in bold. The compounds include a polyphenol variety, such as hydroxybenzoic acids (e.g., gallic acid (1), syringic acid (8)), hydroxycinnamic acids (e.g., chlorogenic acid (4), *p*-coumaric acid (10)), flavanols (e.g., catechin (9)), flavonols (e.g., rutin (12), quercetin 3-*O*-glucoside (13), quercetin (17), kaempferol (18)), and dihydrochalcones (e.g., phloretin dihexoside (14)). These polyphenols exhibit diverse structures that contribute to their antioxidant and anti-inflammatory properties [41,42].

Compound 1 displayed an [M-H]^−^ ion at *m*/*z* 169 and a base peak ion at *m*/*z* 125, corresponding to the loss of CO_2_. The product ions at *m*/*z* 151 [M-H-H_2_O]^−^, *m*/*z* 141 [M-H-CO]^−^, and *m*/*z* 97 [M-H-CO_2_-CO]^−^ suggested a linkage with a carboxylic acid and a phenolic group. In agreement with the literature data, this compound was identified as gallic acid [43]. Compound 8 showed an [M-H]^−^ ion at *m*/*z* 197 and a base peak ion at *m*/*z* 153 [M-H-CO_2_]^−^. The fragment ions at *m*/*z* 179 [M-H-H_2_O]^−^, *m*/*z* 169 [M-H-CO]^−^, and *m*/*z* 161 [M-H-2H_2_O]^−^ agreed with the presence of a carboxylic acid and a phenolic group. Therefore, compound 8 was annotated as syringic acid [32]. Compound 4 displayed a [M-H]^−^ ion at *m*/*z* 353 and was annotated as a caffeoylquinic acid. The product ions at *m*/*z* 191 [M-H-CA]^−^ and *m*/*z* 179 [M-H-QA]^−^ matched with quinic acid and caffeic acid ions, respectively. The identification of the correct caffeoylquinic acid regioisomer was supported by comparison with analytical standards. Therefore, compound 4 was identified as chlorogenic acid [44]. Compound 10 revealed an [M-H]^−^ ion at *m*/*z* 163 and a base peak ion at *m*/*z* 119 [M-H-CO_2_]^−^. The product ions at *m*/*z* 145 [M-H-H_2_O]^−^, *m*/*z* 135 [M-H-CO]^−^, and *m*/*z* 101 [M-H-H_2_O-CO_2_]^−^ indicated the presence of a hydroxycinnamic acid group and a phenolic moiety. Based on the literature data, compound 10 was identified as p-coumaric acid [45]. A flavanol monomer (compound 9) was identified with an [M-H]^−^ ion at m/z 289. The base peak ion at *m*/*z* 245 [M-H-C_2_H_4_O]^−^ and the parent ions at *m*/*z* 271 [M-H-H_2_O]^−^ and *m*/*z* 205 [M-H-C_4_H_4_O_2_]^−^ were in accordance with the literature data. Flavanol monomer identity was verified by comparison with an external standard. Consequently, compound 9 was identified as catechin [46]. Compound 11 showed an [M-H]^−^ ion at *m*/*z* 609 and a base peak ion at *m*/*z* 301 [M-H-Rha-Hex]^−^. The fragment ions at *m*/*z* 463 [M-H-Rha]^−^ and *m*/*z* 179 [M-H-Rha-Hex-C_7_H_6_O_2_]^−^ suggested a linkage between a rutinoside group with aglycone quercetin moiety. Therefore, compound 12 was first identified as rutin [47], and the identity was then confirmed by comparison with an external standard. Compound 13 displayed an [M-H]^−^ ion at *m*/*z* 463. Its tandem mass spectrum was characterized by a base peak ion at *m*/*z* 301 [M-H-Hex]^−^ and a fragment ion at *m*/*z* 179 [M-H-Hex-C_7_H_6_O_2_]^−^, which suggested a quercetin-like scaffold linked to a hexoside group. Based on the literature data and comparison with an external standard, compound 13 was annotated as quercetin 3-*O*-glucoside. Compound 17 showed an [M-H]^−^ ion at *m*/*z* 301 and a base peak ion at *m*/*z* 283 [M-H-H_2_O]^−^, due to the neutral loss of water. Its tandem mass spectrum was characterized by two parent ions at *m*/*z* 179 [M-H-C_7_H_6_O_2_]^−^ and *m*/*z* 151 [M-H-C_8_H_6_O_3_]^−^, derived from the RDA fragmentation and the neutral loss of CO. Based on the literature data and comparison with an external standard, compound 17 was identified as quercetin [47]. Compound 18 displayed an [M-H]^−^ ion at *m*/*z* 285 and a base peak ion at *m*/*z* 267 [M-H-H_2_O]^−^, due to the neutral loss of water. Its tandem mass spectrum was characterized by two parent ions at *m*/*z* 179 [M-H-C_7_H_6_O]^−^ and *m*/*z* 151 [M-H-C_8_H_6_O_2_]^−^, derived from the RDA fragmentation and the neutral loss of CO. Based on the literature data, compound 18 was identified as kaempferol [48]. Compound 14 displayed an [M-H]^−^ ion at *m*/*z* 597. The base peak ion at *m*/*z* 477 [M-H-C_4_H_8_O_4_]^−^ and the fragment ion at *m*/*z* 417 [M-H-Hex-H_2_O]^−^ suggested the linkage of a hexoside group with the phlorizin moiety. Based on this fragmentation pattern, compound 14 was annotated as phloretin dihexoside [47].

Other chemical components were identified, including organic acids (e.g., quinic acid (2)), polar oxylipins (e.g., hydroxy jasmonic acid *O*-hexoside isomer 1 (3), hydroxy jasmonic acid *O*-hexoside isomer 2 (5), hydroxy jasmonic acid *O*-hexoside isomer 3 (7)), and phytohormones (e.g., abscisyl acid *O*-hexoside (6)). In addition, some unknown compounds were annotated and included in the characterization table given their intense signals in negative acquisition mode in HPLC–HESI–MS/MS analysis.

Compound 2 yielded an [M-H]^−^ ion at *m*/*z* 191 and was identified as quinic acid. The tandem mass spectrum was characterized by a base peak ion at *m*/*z* 173 [M-H-H_2_O]^−^ and a parent ion at *m*/*z* 111 [M-H-2H_2_O-CO_2_]^−^, which were due to the cleavage of a carboxylic acid group and the neutral loss of two molecules of water [48]. Three oxylipin isomers (3, 5 and 7) were detected. Their tandem mass spectra displayed an [M-H]^−^ ion at *m*/*z* 387 and characteristic fragment ions at *m*/*z* 369 [M-H-H_2_O]^−^ and *m*/*z* 225 [M-H-Hex]^−^, due to the neutral loss of the hexoside group. Based on the literature data, the three isomers were identified as hydroxy jasmonic acid *O*-hexoside [32]. Compound 6 was detected as formate adduct with an [M+HCOO]^−^ ion at *m*/*z* 471 and was identified as abscisyl acid *O*-hexoside. The fragmentation pattern showed a base peak ion at *m*/*z* 425 [M-H]^−^ and the parent ions at *m*/*z* 263 [M-H-Hex]^−^ and *m*/*z* 219 [M-H-Hex-CO_2_]^−^. The fragmentation suggested the linkage of the apocarotenoid scaffold with the hexoside group. Therefore, the absence of a neutral loss of CO_2_ suggested that the sugar was linked with a glucosyl ester at the aglycon group [32].

**Table 1:** Polyphenols identified or putatively identified by their MS/MS spectra according to the literature by HPLC–MS analysis in negative mode acquisition. Hex, Glu, Rha, HCOOH, CA, and QA indicate hexose, glucose, rhamnose, formic acid, caffeic acid, and quinic acid moieties, respectively. * The identification of these molecules was confirmed using commercial standard.

No.	Compound	R_t_	*m*/*z*	Charge	Fragmentation	Ref.
1	Gallic acid *	2.14	169.80	[M-H]^−^	151.75 [M-H-H_2_O]^−^; 141.84 [M-H-CO]^−^; 125.79 [M-H-CO_2_]^−^; 97.71 [M-H-CO_2_-CO]^−^	[43]
2	Quinic acid	2.17	190.95	[M-H]^−^	172.86 [M-H-H_2_O]^−^; 154.91 [M-H-2H_2_O]^−^; 146.82 [M-H-H_2_O-CO]^−^, 110.96 [M-H-2H_2_O-CO_2_]^−^	[48]
3	Hydroxy jasmonic acid *O*-hexoside isomer 1	4.31	387.18	[M-H]^−^	369.40 [M-H-H_2_O]^−^, 225.02 [M-H-Hex]^−^, 207.00 [M-H-Hex-H_2_O]^−^, 163.10 [M-H-Hex-H_2_O-CO_2_]^−^	[32]
4	Chlorogenic acid *	4.58	353.04	[M-H]^−^	335.12 [M-H-H_2_O]^−^; 191.04 [M-H-CA]^−^; 179.04 [M-H-QA]^−^; 173.05 [M-H-CA-H_2_O]^−^	[44]
5	Hydroxy jasmonic acid *O*-hexoside isomer 2	5.35	387.24	[M-H]^−^	369.26 [M-H-H_2_O]^−^, 225.03 [M-H-Hex]^−^, 207.03 [M-H-Hex-H_2_O]^−^, 163.12 [M-H-Hex-H_2_O-CO_2_]^−^	[32]
6	Abscisyl acid *O*-hexoside	5.37	471.27	[M+HCOO]^−^	425.14 [M-H]^−^; 407.04 [M-H-H_2_O]^−^; 309.05 [M+HCOO-Hex]^−^; 263.13 [M-H-Hex]^−^	[32]
7	Hydroxy jasmonic acid *O*-hexoside isomer 3	6.08	387.16	[M-H]^−^	369.19 [M-H-H_2_O]^−^, 225.03 [M-H-Hex]^−^, 207.15 [M-H-Hex-H_2_O]^−^, 162.93 [M-H-Hex-H_2_O-CO_2_]^−^	[32]
8	Syringic acid *	6.22	197.01	[M-H]^−^	178.92 [M-H-H_2_O]^−^; 168.92 [M-H-CO]^−^; 160.87 [M-H-2H_2_O]^−^; 153.04 [M-H-CO_2_]^−^	[32]
9	Catechin *	6.38	289.09	[M-H]^−^	271.02 [M-H-H_2_O]^−^; 245.05 [M-H-C_2_H_4_O]^−^; 205.03 [M-H-C_4_H_4_O_2_]^−^; 151.04 [M-H-C_7_H_6_O_3_]^−^	[46]
10	*p*-Coumaric acid *	7.12	162.84	[M-H]^−^	145.09 [M-H-H_2_O]^−^; 134.89 [M-H-CO]^−^; 118.97 [M-H-CO_2_]^−^; 101.01 [M-H-H_2_O-CO_2_]^−^	[45]
11	Unknown compound 1	7.52	581.44	[M-H]^−^	419.27; 401.29; 247.22; 233.07	
12	Rutin *	7.83	609.21	[M-H]^−^	591.28 [M-H-H_2_O]^−^; 463.17 [M-H-Rha]^−^; 301.04 [M-H-Rha-Glu]^−^; 179.02 [M-H-Rha-Glu-C_7_H_6_O_2_]^−^	[47]
13	Quercetin 3-*O*-glucoside *	7.96	463.23	[M-H]^−^	445.16 [M-H-H_2_O]^−^; 301.03 [M-H-Glu]^−^; 272.89 [M-H-Glu-CO]^−^; 179.00 [M-H-Glu-C_7_H_6_O_2_]^−^	[38]
14	Phloretin dihexoside	8.17	597.31	[M-H]^−^	477.14 [M-H-C_4_H_8_O_4_]^−^; 459.21 [M-H-C_4_H_8_O_4_-H_2_O]^−^; 417.12 [M-H-Hex-H_2_O]^−^; 399.12 [M-H-Hex-2H_2_O]^−^	[47]
15	Unknown compound 2	8.87	673.36	[M-H]^−^	631.35; 507.14; 313.08; 239.07	
16	Unknown compound 3	10.03	647.43	[M-H]^−^	629.41; 599.73; 443.33; 345.08	
17	Quercetin *	10.24	300.91	[M-H]^−^	283.12 [M-H-H_2_O]^−^; 232.98 [M-H-C_3_O_2_]^−^; 178.91 [M-H-C_7_H_6_O_2_]^−^; 150.94 [M-H-C_8_H_6_O_3_]^−^	[47]
18	Kaempferol	11.57	285.11	[M-H]^−^	267.19 [M-H-H_2_O]^−^; 190.94 [M-H-C_6_H_6_O]^−^; 178.99 [M-H-C_7_H_6_O]^−^; 150.86 [M-H-C_8_H_6_O_2_]^−^	[47]
19	Unknown compound 4	15.80	595.45	[M-H]^−^	467.33; 415.22; 315.01; 258.97	
20	Unknown compound 5	21.03	555.40	[M-H]^−^	537.40; 491.35; 449.10; 225.07	
21	Unknown compound 6	22.36	666.12	[M-H]^−^	666.17; 636.12; 591.58; 481.03	

#### 3.1.2. Quantitative Polyphenol Analysis

The quantitative polyphenol analysis of *A. unedo* L. pomace hydroalcoholic extract was performed by HPLC–DAD–FLD (Table 2). Six polyphenols were quantified, and the results were expressed as mg/g DW. Given the limited exploration of *A. unedo* L. pomace in the literature, making comparisons proves challenging. Moreover, our results agreed with those reported by other authors for polyphenol-enriched extracts obtained from *A. unedo* L. fruits. Ganhão et al. [49] reported total hydroxybenzoic acid and hydroxycinnamic acid contents of 1.122 ± 0.094 and 0.0010 ± 0.0001 mg/g DW, respectively. Our results displayed similar values, with a gallic acid content of 2.440 ± 0.080 mg/g DW as hydroxybenzoic acid, and chlorogenic acid and p-coumaric acid contents of 0.003 ± 0.001 mg/g DW and 0.020 ± 0.004 mg/g DW as hydroxycinnamic acids. On the other hand, some significant differences were observed in flavonol contents. Ganhão et al. reported a total flavonol content of 0.036 ± 0.004 mg/g DW, which is significantly lower than our quantification, which revealed a quercetin 3-*O*-glucoside and quercetin content of 0.007 ± 0.001 mg/g DW and 0.160 ± 0.030 mg/g DW, respectively. This difference may be due to the different polyphenol content in *A. unedo* L. pomace compared to the fruits. Moreover, Rodrigues et al. reported that the fermentation and/or distillation process elevated the total flavonoid content in *A. unedo* L. pomace residue by twofold compared to the fruits [24]. In addition, another relevant difference is related to the extraction solvents and approaches reported by the authors, which resulted in different extractability of phenolic compounds from the plant matrix. In fact, the authors performed the extraction using an acetone/water mixture with accelerated solvent extraction (ASE), an innovative approach based on a pressurized liquid extraction. Although this method is a novel green extraction approach, ASE requires stringent validation of the extraction parameters (e.g., polarity and volume of extracting solvent, sample size, temperature, pressure, and extraction cycle) for an exhaustive polyphenol recovery [50]. Therefore, the lack of ASE parameter optimization could explain the reduced content of some polyphenols compared with those reported in our study. In fact, our extraction protocol was based on ultrasound-assisted extraction (UAE) and was previously validated for the extraction of several polyphenol classes with recovery values close to 100% [32].

Ultrasound-assisted extraction (UAE) enhances the recovery of polyphenols compared to conventional methods by improving mass transfer and cell disruption through acoustic cavitation [51]. This phenomenon facilitates the release of bioactive compounds, reducing extraction time and solvent consumption while increasing yield. UAE operates at lower temperatures, minimizing the degradation of thermolabile polyphenols and preserving their bioactivity [52]. Additionally, its compatibility with green solvents aligns with sustainable extraction principles, making it an efficient and environmentally friendly alternative to traditional techniques such as maceration or Soxhlet extraction [53].

The high concentrations of gallic acid and flavonoids, such as quercetin 3-*O*-glucoside, identified in the *A. unedo* L. pomace extract suggest its potential for cosmetic applications. These compounds demonstrate antioxidant, photoprotective, and anti-aging properties, which can reduce skin pigmentation, counteract UV-induced damage, and promote skin health while preventing premature aging. This aligns with the findings of Habachi et al. [54], which highlight the phenolic richness of *A. unedo* and its promising role in developing innovative active ingredients for cosmetics and dermatology.

### 3.2. Total Polyphenol Content and Antioxidant Activity

As highlighted earlier, polyphenols are distinguished by their notable antioxidant potential, a trait particularly relevant in the cosmetics industry due to their efficacy in mitigating reactive oxygen species, thereby addressing concerns such as premature aging. Consequently, the present study delved into assessing the total polyphenol content (TPC) by FOLIN assay and the antioxidant capacity of *A. unedo* L. pomace utilizing DPPH, FRAP, and ABTS methodologies. The TPC yielded a value of 14.04 ± 0.49 mg GAE/g DW (Table 3). As was previously mentioned, only one investigation utilizing a supercritical fluid extraction technique has explored the extraction of anthocyanins from *A. unedo* pomace [23], with the rest focusing on fresh *A. unedo* fruit. For example, Albuquerque et al. [55] optimized a method for the extraction of polyphenols from fresh *A. unedo* fruits using the USE technique, yielding a total polyphenol concentration of 64.61 ± 0.91 mg/g dw under optimal conditions. El Cadi et al. [56] also evaluated total polyphenol concentrations in *A. unedo* fruits, employing solid–liquid extractions with two different solvents (MeOH and ethyl acetate (EtOAc)) following of sonication and achieving the highest concentration with the mixture of MeOH: H_2_O (4:1), with an extraction yield of 75.88 ± 3.1 mg/g DW. As can be observed, an expected decrease in concentration was detected from the fresh fruit, based on the distillation process the fruit went through before the elaboration of the beverage. However, a high content of phenolic compounds was demonstrated in the pomace, even after undergoing the distillation process.

**Figure 2:**
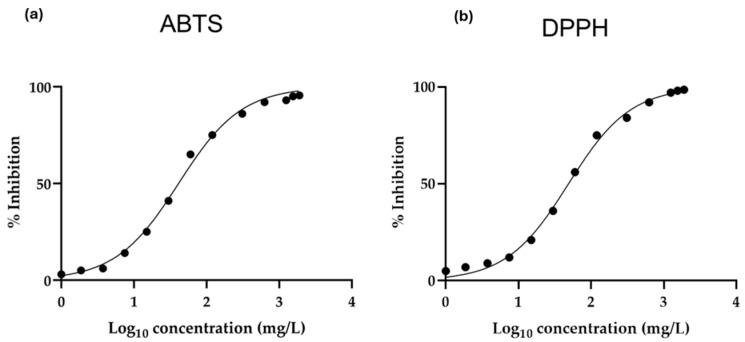
Antiradical activity of *A. unedo* L. pomace extract, expressed as (**a**) IC_50_ of the ABTS assay and (**b**) IC_50_ of the DPPH assay. Values represent the mean ± standard deviation of triplicate reading.

Therefore, the next step was to demonstrate the antioxidant properties associated with phenolic compounds in the pomace in order to test their possible application in cosmetic formulations. The DPPH methodology yielded an antioxidant capacity of 110.67 ± 0.88 μmol TE/g DW, the FRAP methodology resulted in 122.16 ± 1.77 μmol TE/g DW, and the ABTS methodology recorded 151.94 ± 0.45 μmol TE/g DW (Table 3). Last, IC_50_ was calculated for the DPPH and ABTS assays. Different dilutions of *A. unedo* L. pomace extracts were prepared at the following concentrations: 1, 1.87, 3.75, 7.5, 15, 30, 60, 120, 310, 630, 1250, 1550, and 1900 mg/L. The percentage of inhibition for the two assays was measured for each concentration, and an inhibition curve was obtained (Figure 2). In the case of the DPPH methodology, the IC_50_ obtained was 49.49 mg/L, with a R^2^ of 0.9463. In the case of the ABTS methodology, the IC_50_ obtained was 42.25 mg/L, with a R^2^ of 0.9544.

Given the limited exploration of *A. unedo* L. pomace in the literature, making comparisons proves challenging. Nevertheless, our findings align with observations made by Duarte et al. and Alburquerque et al. [28,55] concerning the antioxidant capacity of this residue. Hence, the demonstrated antioxidant potential of the extracts underscores the presence and efficacy of phenolic compounds within this sample. Furthermore, a comparative analysis was conducted of the antioxidant capacity values obtained for *Arbutus unedo* L. pomace with those of other agro-industrial by-products described in the literature. Sandoval-Cárdenas et al. [57] evaluated the concentration of bioactive compounds and antioxidant capacity of hydrolyzed corn cob residues, which showed a concentration of 5.36 mg rutin equivalent/mL in the DPPH assay and 10.7 mg Trolox equivalent (TE)/mL in the ABTS assay. In contrast, non-hydrolyzed corn cob exhibited significantly lower concentrations of 1.28 mg/mL and 1.64 mg/mL in the aforementioned assays. Conversely, Chaudhry et al. [58] evaluated the antioxidant potential of banana peels, demonstrating a value of 82.5 mg TE/g for the DPPH method. In contrast, a study carried out by Abbasi-Parizad et al. [59] showed that grape pomace by-products had a concentration of 87.8 μM TE/g and that spent coffee by-products had a concentration of up to 89.8 μM TE/g. In contrast, tomato pomace had a concentration of 21.5 ± 0.0 μM. Finally, Sánchez et al. [60] evaluated the antioxidant activity of cupuaçu (*Theobroma grandiflorum*) seeds, which showed an ABTS value of 151.0 ± 5.5 mg TE/100 g for the extract and a DPPH value of 85.4 ± 1.7 mM TE/L.

The results collected from the literature position *A. unedo* pomace as a promising antioxidant source, comparable or superior to the activity of various agro-industrial wastes. This supports its potential application in food, nutraceutical, and pharmaceutical formulations.

Regarding the cosmetics industry, the extracts derived from the *A. unedo* L. pomace exhibited considerable antioxidant potency, particularly when juxtaposed with numerous agricultural and food industry by-products currently under consideration for integration into cosmetic formulations, such as virgin Calophyllum, Campomanesia fruit peels, milk thistle fruits, or broccoli by-products [61,62,63,64,65].

### 3.3. Cell Responses to In Vitro Treatments

**Figure 3:**
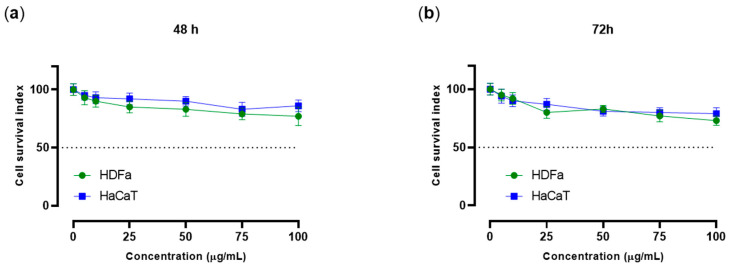
Preclinical bioscreens for the evaluation of *A. unedo* L. pomace extract safety and biocompatibility in human skin models. Concentration–response curves by the “cell survival index” for HDFa and HaCaT cells following 48 h (**a**) and 72 h (**b**) of treatment with a range of concentrations (5–100 μg/mL) of *A. unedo* L. pomace extract. Results are expressed in line graphs as a percentage of untreated control cells and are reported as the mean of three independent experiments ± SEM (*n* = 15).

To explore the biocompatibility as well as the safety profile of the *A. unedo* L. pomace extract by preclinical investigations, we performed targeted in vitro bioscreens on human skin cell models, i.e., immortalized keratinocytes (HaCaT) and dermal fibroblasts (HDFa). The extract was tested using concentrations ranging from 5 to 100 μg/mL throughout incubations of 48 and 72 h. The obtained data, reported as “cell survival index” in Figure 3 by concentration–effect curves, showed no significant variations after treatments in vitro compared to untreated cells. No biological effects or interference with cell viability and proliferation were observed even at the highest concentrations (100 μg/mL) following long exposure times (72 h). Thus, these results suggest a good safety profile for *A. unedo* L. pomace extract, providing evidence for its biocompatibility in the skin system.

### 3.4. Antioxidant Activity In Vitro

**Figure 4:**
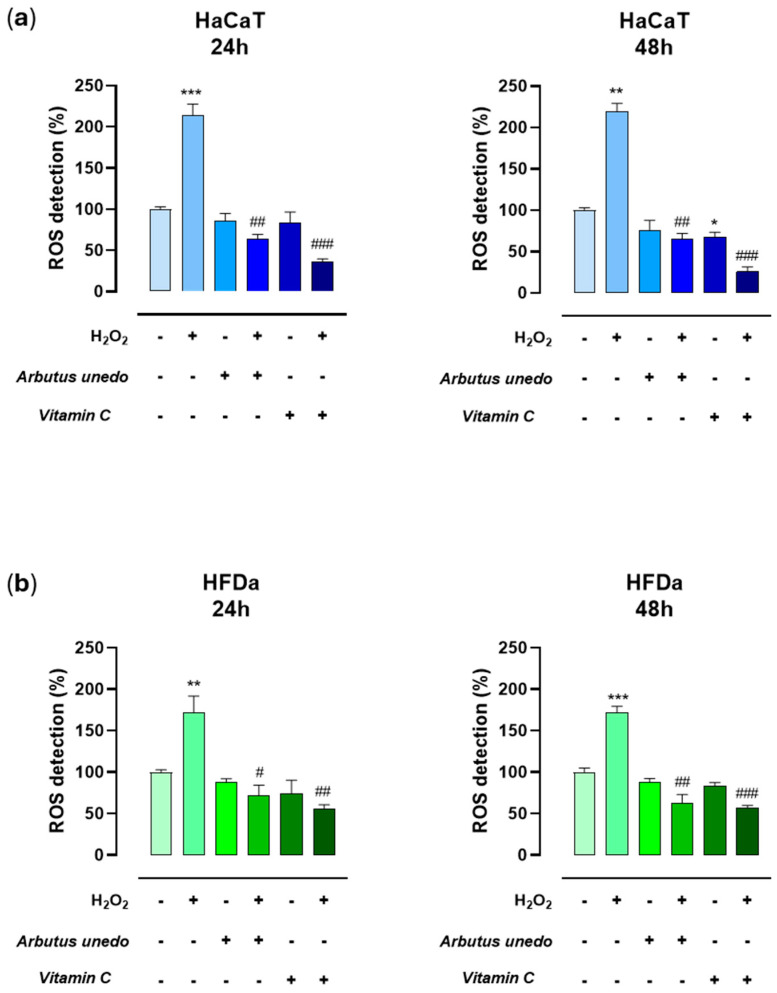
Antioxidant activity of *A. unedo* L. pomace extract in human skin models. ROS detection in HaCaT (**a**) and HDFa (**b**) after 24 h and 48 h of treatment with H_2_O_2_ alone or in combination with 50 μg/mL of *A. unedo* L. pomace extract, or with vitamin C. Results are expressed as a percentage of untreated control cells and are reported as the mean of three independent experiments ± SEM (*n* = 15). * *p* < 0.05; ** *p* < 0.01; *** *p* < 0.001 vs. control; ^#^ *p* < 0.05, ^##^ *p* < 0.01; ^###^ *p* < 0.001 vs. H_2_O_2_-treated cells.

To explore *A. unedo* L. pomace extract antioxidant activity in cells, we evaluated its ability to reduce ROS formation and oxidative stress in preclinical models of human skin. As described in the experimental section, HDFa and HaCaT cells were exposed to H_2_O_2_ to induce oxidative stress, and then treated for 24 h or 48 h with different concentrations (25, 50, and 100 μg/mL) of the extract or with 50 µg/mL of vitamin C. Significant antioxidant protection by *A. unedo* L. pomace extract was observed in both epidermal and dermal cells in all the experimental conditions used. However, the biological effects we detected were independent of the extract concentration and contact times. Figure 4 shows the antioxidant effects at the intermediate extract concentration of 50 µg/mL, serving as a representative biological trend. Moreover, the extract’s ability to reduce ROS formation was similar to that of vitamin C application, proving the protective antioxidant role of the *A. unedo* L. pomace extract. Interestingly, the extract and vitamin C showed the same biological behavior in vitro, not being able to decrease ROS production in basal conditions but significantly counteracting hydrogen peroxide-dependent oxidative stress. This outcome can have a significant biological impact since high cytosolic ROS levels are typically involved in extensive biomolecular damage to DNA, proteins, and lipids, which can in turn be related to cell death and skin-aging processes. As well as the direct scavenging action of radical species, the antioxidant protection of natural polyphenols may also involve the activation of heterodimers of the NF-E2-related factor 2 (Nrf2)/antioxidant responsive element (ARE) pathway. Indeed, this pathway is an important antioxidant cellular defense mechanism that acts by regulating the expression of target genes implicated in the maintenance of redox homeostasis. Since many natural polyphenols have recently emerged as Nrf2 activators, it cannot be excluded that bioactive components of the *A. unedo* L. pomace extract may act through this mechanism [66].

## 4. Conclusions

In this study, we chemically and biologically characterized the pomace extract of *A. unedo* L. It was demonstrated to contain a notable quantity of polyphenolic compounds and exhibited robust antioxidant properties both in test tubes and in vitro. To continue, the extract also exhibited excellent biocompatibility in human skin cells, thereby reinforcing its potential as a safe and effective ingredient.

Based on these results, we recognize the potential of this extract and esteem its promising role in supporting skin health and promoting its longevity. Indeed, the extract’s ability to reduce ROS levels in the presence of oxidative stress highlights its potential in counteracting aging and safeguarding against environmental damage.

Industrial-scale valorization of *A. unedo* L. pomace faces some technical and economic challenges. From a technical point of view, it is necessary to study how large-scale fermentation and distillation conditions can affect the final composition of bioactive compounds. However, several previous studies have demonstrated the possibility of extracting these compounds on an industrial scale.

From an economic perspective, large-scale production requires substantial investment in infrastructure, including specialized extraction and purification facilities, as well as ensuring compliance with regulatory standards for cosmetic ingredients. However, this could be overcome by establishing strategic relationships with distilleries and cosmetics manufacturers to ensure a continuous and cost-effective supply of raw materials, not to mention that such raw materials are currently being disposed of at virtually zero cost. In conclusion, *A. unedo* L. pomace extract is a promising natural alternative to synthetic antioxidants in dermatological and pharmaceutical formulations. It is rich in polyphenolic compounds with strong antioxidant properties, comparable to vitamin C, and effectively reduces oxidative stress in skin cells. The extract is safe, biocompatible, and supports skin health by counteracting aging and environmental damage. While industrial-scale production poses challenges, its sustainable use aligns with circular economy principles, making it a viable and eco-friendly ingredient for skincare and pharmaceutical applications.

## 5. Limitations

Overall, these findings lend support to further investigation and utilization of *A. unedo* L. pomace as an active ingredient. Forthcoming research should go deeper into the investigation of other molecular and cellular pathways on skin cells, formulation optimization, and clinical trials in order to fully exploit the potential benefits of this promising by-product.

## Figures and Tables

**Table 2:** Quantitative analysis of polyphenol content. Results are expressed as mean ± SD.

Compound	Content (mg/g DW)
Gallic acid	2.440 ± 0.080
Catechin	0.210 ± 0.005
Chlorogenic acid	0.003 ± 0.001
*p*-Coumaric acid	0.020 ± 0.004
Quercetin 3-*O*-glucoside	0.007 ± 0.001
Quercetin	0.160 ± 0.030

**Table 3:** Total polyphenol content and antioxidant activity of *A. unedo* L. pomace extract. Values represent the mean ± standard deviation of triplicate reading.

	Content (Mean ± SD)
Total polyphenol content (TPC)	14.04 ± 0.49 mg GAE/g DW
DPPH	110.67 ± 0.88 μmol TE/g DW
ABTS	122.16 ± 1.77 μmol TE/g DW
FRAP	151.94 ± 0.45 μmol TE/g DW

## Data Availability

Data presented are contained within the article.
